# Ways to Improve Multitasking: Effects of Predictability after Single- and Dual-Task Training

**DOI:** 10.5334/joc.142

**Published:** 2021-01-07

**Authors:** Harald Ewolds, Laura Broeker, Rita F. de Oliveira, Markus Raab, Stefan Künzell

**Affiliations:** 1 University of Augsburg, Augsburg, Germany; 2 German Sport University Cologne, Cologne, Germany; 3 London South Bank University, London, UK

**Keywords:** Action and perception, Implicit learning, Visual perception, Auditory processing

## Abstract

In this study we investigated the effect of predictability on dual-task performance in two experiments. In the first experiment 33 participants separately practiced a continuous tracking task and an auditory reaction time task. Both tasks had a repeating element that made them predictable; in the tracking task this was a repeating segment, and in the auditory task this was an auditory sequence. In addition, one group obtained explicit knowledge about the repeating sequence in the tracking task while the other group trained implicitly. After training, single- and dual-task performance was tested at a post test and retention test. Results showed that predictability only improved performance in the predictable tasks themselves and dual-task costs disappeared for the tracking task. To see whether the task-specific effect of predictability was the results of task prioritization, or because task representations did not have much chance to interact with each other, we conducted a second experiment. Using the same tasks as in Experiment 1, 39 participants now trained both tasks simultaneously. Results largely mirrored those of the first experiment, demonstrating that freed-up resources due to predictability in one task could not be re-invested to improve in the other task. We conclude that predictability has a positive but task-specific effect on dual-task performance.

## INTRODUCTION

Switching between tasks in complex work environments, dividing attention between cellphones and TVs or simultaneously handling complex car cockpits and driving – multitasking is omnipresent in modern society. In lab settings, performing multiple tasks simultaneously reliably leads to dual-task costs which are most often explained by structural or strategic bottlenecks (Meyer & Kieras, 1997; Pashler, 1994), capacity limitations (Damos & Wickens, 1980; Kahneman, 1973; Navon & Gopher, 1979; Wickens, 2008) or cross-talk (Navon & Miller, 1987; Swinnen & Wenderoth, 2004). However, some studies have seen reduced or even eliminated dual-task costs, either after large amounts of training (Ruthruff et al., 2006), or by using ideomotor-compatible tasks (Halvorson et al., 2013; Halvorson & Hazeltine, 2015). Künzell et al. (2018) argued that both large amounts of practice and ideomotor compatibility, make tasks more predictable which can facilitate automatic processing and reduce interference between tasks. Previous studies indeed found a beneficial effect of making one of two tasks predictable, although performance improvements were only visible in the predictable task. For instance, adding predictability to a tracking task improved tracking accuracy when simultaneously performed with an auditory reaction time task, but reaction times did not improve (Broeker et al. submitted; Ewolds, Broeker, Oliveira, Raab, & Künzell, 2017). To further elucidate the role of predictability in multitasking, Experiment 1 of our study aimed to elaborate on these previous findings. We investigated whether the simultaneous performance of two predictable tasks, as opposed to just one predictable task as in previous studies, would enhance dual-task performance in both tasks after single-task training on both tasks. Following the findings of Experiment 1, which indicated only task-specific effects of predictability, we decided to conduct Experiment 2, in which participants did dual-task training.

In motor control, the expected sensory consequences are predicted by forward models (Wolpert et al., 1995). When going down a familiar set of stairs (as a proficient walker) or a well-known ski run (as an expert skier), there is a match between expected consequences and actual sensory feedback. If there is no mismatch, movement is controlled with minimal awareness and attention. However, if a mismatch occurs, be it due to an error in the forward model or the unpredictability of the situation, attentional processes will intervene (Frith et al., 2000; Neumann, 1984). Attentional processes require resources, which are limited (Wickens, 2008), therefore increased predictability should reduce resource requirements. Predictability of a task during dual-tasking may be desirable not just because that task itself becomes less resource dependent, but also because performance of the other task might benefit from the freed-up resources.

Whereas the above arguments are theoretically convincing, empirical evidence is less clear. For instance, Ewolds et al. (2017) found the beneficial effects of predictability to be contained in the predictable task; the resources freed up by increased predictability were not invested into the other task. A possible explanation for this is that resource allocation strategy may depend on specific task characteristics (Tombu & Jolicœur, 2003) rather than just available resources. It is possible that the predictable tracking task in Ewolds et al. (2017) prevented resources to be carried over to the reaction time task, because a tracking task requires continuous execution. In this tracking task a moving target had to be followed with a joystick as closely as possible. Since it is impossible to take a break in a tracking task without a visible increase in error it demands resources continuously, shifting priority to that task. Second, Navon and Gopher (1979) argued that when there is only one limited pool of general resources, humans employ utility considerations to decide on economic allocation of limited resources, so not reinvesting into another task might satisfy this. Third, making a task predictable may have a side effect of making that task more salient in the course of an experiment. Therefore, the resources freed up in the tracking task might all have been reinvested into the tracking task itself, and not, as the equal-priority instructions were meant to achieve, distributed equally over both tasks. This raises the question of what would happen if both tasks are made predictable.

Prior knowledge can make a situation predictable. For instance, in tennis knowledge of the usual motion of a tennis ball in combination with visual information will tell a practiced player in advance where the ball will arrive (Körding & Wolpert, 2006). To address the question of how this form of predictability might influence multitasking performance, we investigated the effect of predictable and unpredictable movement sequences, as has often been done in tracking studies (Künzell et al., 2016; Pew, 1974; Wulf & Schmidt, 1997) and in serial reaction time (SRT) tasks (Curran & Keele, 1993). In tracking tasks participants typically follow a target that in a certain segment of the screen (i.e. first third, second third or last third) always has the same movement pattern across trials, whereas the other segments contain random movement patterns every trial. Similarly, in SRT tasks participants are instructed to press buttons in response to cues on a screen which, for the majority of the time, follow a repeating order. Typically, participants perform better on repeating segments and orders after some practice compared to random segments or orders. This happens without subjects being aware of the repetition, therefore these tasks provide an excellent paradigm to study implicit learning.

Implicit learning and explicit learning may both lead to a mostly implicit knowledge base over time, as many studies in motor learning have shown (Kal et al., 2018), but in the beginning of practice different knowledge bases can be identified. Via questionnaires the absence of explicit knowledge can be established which may indicate a largely implicit knowledge base (Schmidtke & Heuer, 1997). The absence of implicit knowledge is difficult to measure, so the degree to which explicitly instructed participants also build an implicit knowledge base cannot be determined, although it is often assumed that learning shown in the presence of a dual task is an indication of implicit learning (Curran & Keele, 1993; but see Shanks, Rowland, & Ranger, 2005). Explicitly instructed participants benefit directly from explicit knowledge about regularities in an SRT task while implicitly learning participants are slower but achieve better performance on retention tests. Both implicit and explicit knowledge about regularities are a basis of anticipatory control of perception and action. Data from previous experiments confirmed the idea that both types of knowledge aid dual-task performance since both unaware and aware participants benefited from repeating sequences (Ewolds et al., 2017). In contrast, many studies employing more complex movements (with tracking probably lying somewhere between the SRT task and most sports in terms of complexity) report that for experts in particular explicit knowledge hampers performance, presumably because it interferes with well-learned ‘automatized’ processes (Beilock et al., 2002; Koedijker et al., 2011; Masters, 1992; Poolton et al., 2006). It has been argued that the attentional demands during dual-task learning favor implicit learning and may even prohibit explicit learning, leading to better performance of implicitly instructed participants (Berry, 1993; Maxwell et al., 2003). The current study examines both implicitly and explicitly instructed participants to test whether implicitly instructed participants have an advantage in dual-task situations.

In the first experiment we tested the effect of predictability on dual-tasking with both a predictable tracking task and a predictable auditory reaction time task. The tracking task comprised of two outer random segments and a repeating segment in the middle; the auditory task across the full trials involved either a repetitive tone sequence or random tones. Both tasks were practiced and learned as single tasks and only later tested as a dual task. If both tasks draw on a single resource and if predictability influences prioritization of both tasks equally, both tasks should benefit from predictability in the other task. Alternatively, if both tasks draw on separate resources predictability in one task might only enhance processing for this particular task, but not for the other task.

## EXPERIMENT 1

### MATERIALS AND METHODS

#### Participants

Seven participants dropped out during the testing, leaving 33 university students (18 female). The *implicit learning group* had 17 participants (*M* = 25.0 years, *SD* = 2.2) and the *explicit learning group* had 16 participants (*M* = 25.1 years, *SD* = 2.8). A previous study found an effect size of η_p_^2^ = .365 when comparing performance on repeating segments with random segments (Ewolds et al., 2017). Based on this a G*Power (Version 3.1.9.2) analysis revealed a test power of .95 and a required sample size of 14 participants (effect size f = 0.76, α = .05, 1–β = .95). All participants reported normal or corrected-to-normal vision and no neurological disorders. Participants gave informed consent prior to the start of the experiment and received payment of 20 € after completing the experiment. The research was approved by the local ethics committee.

#### Experimental setup

Participants sat at a table with their preferred hand controlling a joystick (Speedlink Dark Tornado). At a distance of 40 cm from the joystick the task was displayed on a 24” computer screen (144 Hz, 1920 × 1080 pixel resolution). The tracking program ran on a Windows 7 computer and data was recorded at 120 Hz. The stimuli of the auditory go/no-go task were delivered via Philips SHP2500 stereo headphones and we recorded responses with a foot pedal (f-pro USB-foot switch, 9 × 5 cm). To ensure that tracking performance was not influenced by moving the joystick through the resting zone, which causes an irregularity in resistance, we made sure that the motion required to position the cursor from the upper to the lower edge of the screen fell within the upper half of the range of motion of the joystick on the y-axis.

#### Tasks and display

The tracking task was based on the study by Künzell et al. (2016). The tracking path consisted of three segments. The formula used to create the segments was taken from Wulf & Schmidt (1997):

f\left(x \right)\; = \;{b_0}\, + \,\mathop \sum \limits_{i\, = \,1}^6 {a_i}\,sin\left({i\;\cdot\;x} \right)\; + \;{b_i}\,cos\left({i\;\cdot\;x} \right)

with *a_i_ and b_i_* being a randomly generated number ranging from –4 to 4 and *x* in the range [0, 2π]. For this experiment 41 segments of similar length and number of extrema were selected to prevent learning effects due to differences in difficulty (Chambaron et al., 2006). From the 41 segments available, the path for each participant consisted of a (unique) middle repeating segment and two outer segments selected randomly without placing back from the remaining 40. We chose the outer segments in such a way that each segment occurred an equal amount of time across participants. This meant that each participant would learn a different middle segment while the overall difficulty level was kept equal. For the tracking task, participants tracked a red target square along the invisible curve by controlling a cursor displayed as a white cross (both target and cursor fit within 19 × 22 pixels). Velocity of the target was constant along the curves, ensuring a uniform difficulty level across the trial. Trials lasted between 40 and 44 seconds since Künzell et al. (2016) were best able to find motor learning at those target velocities.

The second task was an auditory go/no-go reaction time task, similar to the second task used in dual-task studies investigating implicit sequence learning in SRTs (e.g. Heuer & Schmidtke, 1996). For the reaction time task participants pressed a pedal for the target tone (Tone A: 1700 Hz, 75 ms) and ignored distractor tones (Tone B: 217 Hz, C; 600 Hz, D: 1086 Hz, 75 ms). On each trial the number of target tones was 9 or 10 and the number of distractor tones was 26 or 27, depending on the length of the track. The duration between tones on random and sequenced trials varied between 700–1200 ms and no tones were placed earlier than 500 ms after the start of the trial or 500 ms before the end of the trial. In the predictable reaction time task tones were always placed in the same order, yielding a very simple repeating sequence of tones (BCADBCADBCAD etc.), whereas in the random tone task the tones were positioned in a random order. Frequency distributions of the four tones were the same for the random and predictable reaction time task.

#### Procedure

After giving informed consent each participant sat at the table and adjusted their seat and pedal. The experimenter explained that the cursor and the target moved automatically from left to right along a sinusoidal curve, and that the goal was to keep the cursor as accurately as possible on the target by moving the joystick forward and backward (movement was coupled to the target along the x-axis). For the auditory reaction time tasks participants were asked to respond as quickly as possible to the target tone (played beforehand) by pressing a pedal with their preferred foot. Participants were told to not respond in anticipation but wait until they heard the target tone. Every five trials feedback about performance on both tasks was given on the screen. Participants were instructed to try their best on both tasks equally.

**Figure 1:**
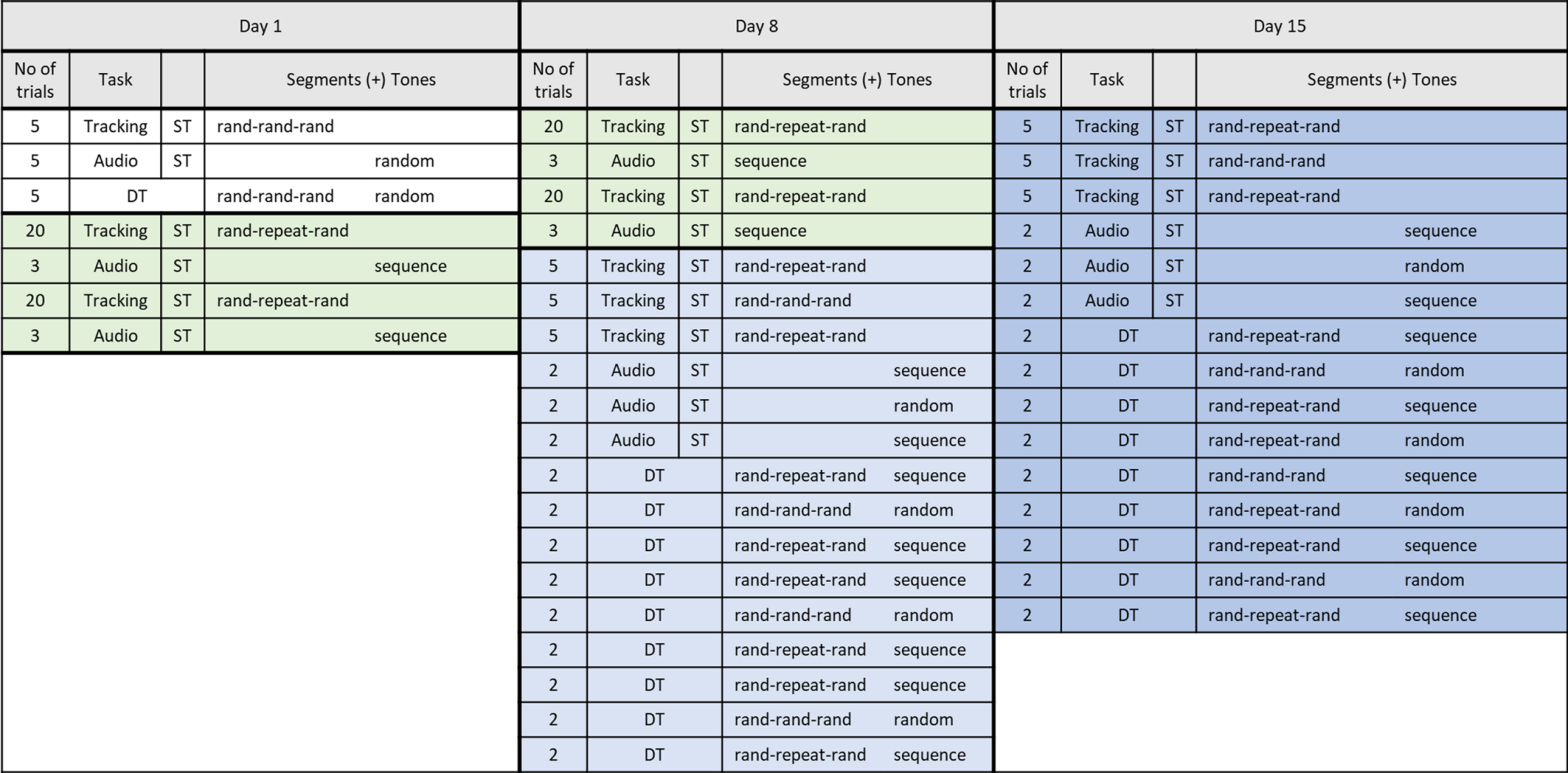
Experimental design for Experiment 1. The pretest was done for familiarization and stimuli were randomized preventing learning. A predictable tracking task contained a repeating middle segment (i.e. rand-rep-rand), an unpredictable tracking tasks contained three random segments (i.e. rand-rand-rand). The predictable audio task was a tone sequence (i.e. sequence), with every fourth tone being the target tone. The unpredictable audio task used the same tones but in a random order (i.e. random). ST = single task, DT = dual-task.

On the first day participants completed five familiarization trials in tracking, the reaction time task and the dual-task, all with random tracking sequences and random tone placement (using the same tones as in later phases). They then completed two tracking task training blocks with a repeating middle segment consisting of 20 trials each. Just before the training blocks, participants in the *explicit learning group* received information that there would be a repeating middle segment in the training blocks (no such instruction was given to the *implicit learning group*). After the tracking training block participants performed three trials of the auditory task, which was enough to induce explicit knowledge of the tone sequence as shown in a pilot. One week later (day 8) they completed two additional training blocks as on day one. At the end of day 8 participants completed a test block of 39 trials in different conditions as displayed in ***Figure 1***. Participants were informed which tasks they had to do but neither group was informed about the occurrence of a repeating middle segments during the test trials. The test block was repeated on a third day in a retention block.

After all blocks were completed, the *implicit learning group* answered a questionnaire to determine how aware they were of the repeating middle segment. The questionnaire contained seven questions designed to gradually probe participants about their knowledge of the repeating middle segment. The questions were: 1) Did you notice anything special during the experiment? 2) Was there something that helped or hindered you while performing the tracking? 3) Did you apply any rules? 4) Did you notice anything special concerning the path of the target? 5) The target followed a certain path. Did you notice any segments in this path? 6) There were three segments in the path, the first, the middle and at the last segment. One of these segments was always repeating? Did you notice? 7) Which segment was the repeating segment, the first, the middle or the last segment?

#### Data analyses

Tracking performance was measured by calculating the root mean square error between the target and user-controlled cursor (RMSE; Wulf & Schmidt, 1997). One RMSE corresponds to about 0.56 cm difference between cursor and target on the screen. We followed recommendations by Zhu et al. (2014) to take the average performance of the two outer segments and compare it against the repeating middle segment. For the auditory go/no-go task we recorded reaction times and errors. Since data revealed that participants made very few errors in the reaction time task, less than 1 error per trials in all conditions, we took RTs as the variable of analysis for the reaction time task.

A prerequisite for the testing of predictability effects is the establishment of learning effects specific to the repeating segment and the predictable tone sequence. To test for the acquisition of such knowledge we submitted training block RMSEs to a 4 × 2 × 2 mixed analysis of variance (ANOVA) with within-subjects factors Block (4 training blocks) and Segment (middle segment vs. random segments), and between-subjects factor Group (implicit vs. explicit). Similarly, learning in the auditory reaction time task was tested with a repeated measures ANOVA with the within-subjects factor Block (4 training blocks). Note that for the reaction time task we cannot establish in this phase how much of the learning is due to general training effects or sequence specific knowledge, since no comparison with a random reaction time task was made.

To test the effect of predictability on RMSEs in the Test block and Retention Block, we ran a 2 × 3 × 2 × 2 mixed ANOVA with the factors Segment (predictable vs. random), Tones (predictable vs. random vs. none – with ‘none’ being single task tracking), Test (Test vs. Retention test) and between-subjects factor Group (implicit vs. explicit).

To test the effect of predictability on reaction times in the Test Block and Retention Block, we ran a 3 × 2 × 2 × 2 mixed ANOVA with factors Segment (none vs. predictable vs. random), with ‘none’ being single task performance, Tones (predictable vs. random), and Block (Test Block vs. Retention Block) and between-subjects factor Group (implicit vs. explicit). Whereas in the Training Blocks the effect of predictability in the tracking task was calculated by comparing performance on the middle segment against performance on the outer segment, in the Test block and Retention Test we always compared middle segments, which contained all possible conditions; predictable or random tracking with no tones, predictable tones or random tones. Significance level was set at *p* < 0.05. Greenhouse-Geisser correction was applied if the sphericity assumption was violated.

### RESULTS

#### Acquisition of predictability-based knowledge

We first established that there were learning effects specific for the repeating segment during the training phase. For the tracking task there was a main effect of Block, *F*(1.81, 56.04) = 9.63, *p* < .001, η_p_^2^ = .237 (Block 1: *M* = 1.64 ± 0.24 cm; Block 4: *M* = 1.50 ± 0.32 cm), showing that participants improved tracking performance across blocks in general, and a main effect of Segment, *F*(1, 31) = 26.42, *p* < .001, η_p_^2^ = .460 (Repeating segment: *M* = 1.51 ± 0.27 cm; Random segment: *M* = 1.60 ± 0.26 cm), showing that participants performed better on repeating compared to random segments. There was no effect of Group, *F*(1, 31) < 1, *p* = .596, and none of the interactions were significant, Block × Segment, *F*(3, 93) = 1.58, *p* = .200, Group × Block, *F*(3, 93) < 1, *p* = .707, Block × Segment × Group, *F*(3, 93) = 1.85, *p* = .144.

For the reaction time task there was also a main effect of Block, *F*(1.74, 53.89) = 7.64, *p* < .001, η_p_^2^ = .198, (Block 1: *M* = 445 ± 73 ms, Block 4: *M* = 399 ± 84 ms), so participants improved reaction times from the first to last training block. There was no effect of Group, *F*(1, 31) < 1, *p* = .436, and no Group × Block interaction, *F*(3, 93) < 1, *p* = .816.

#### Test Block and Retention Block

**Figure 2:**
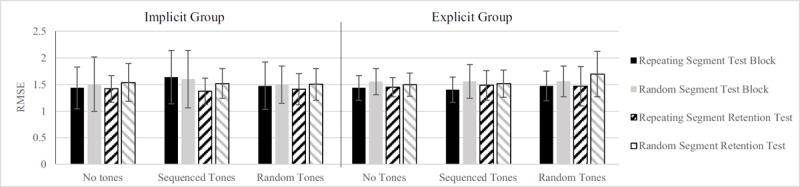
Tracking results from the Test Block and Retention Test in Root Mean Square Error (RMSE). Bars within a cluster show the tracking performance for repeating and random segments per group while the different cluster represent the conditions of the auditory task. We found significantly better tracking on repeating segments, but no differences between groups or tracking with sequenced or random tones. Error bars represent the standard deviation of the mean.

Regarding the tracking task, there was a significant main effect of Segment, *F*(1, 30) = 13.32, *p* = .001, η_p_^2^ = .307 (repeating segment *M* = 1.46 ± 0.32 cm; random segments *M* = 1.55 ± 0.34 cm) on RMSEs, so participants’ tracking performance was better on repeating segments compared to random segments, see ***Figure 2***. There was no effect of Tones, *F*(2, 60) < 1, *p* = .568, or Group, *F*(1, 30) < 1, *p* = .878, nor of Test, *F*(1, 30) < 1, *p* = .432. The failure to find an effect of tones meant that there was no effect on tracking performance whether a random tones or sequenced tones were played. Moreover it means that we found no significant dual-task costs (No tones *M* = 1.48, sequenced tones *M* = 1.51, random tones *M* = 1.51. We found a significant Test × Segment × Group interaction, *F*(1, 30) = 4.67, *p* = .039, η_p_^2^ = .135, which indicated that whereas the implicit learning group improved from Test to Retention on the repeating segment (from *M* = 1.52 cm to *M* = 1.40 cm), the explicit learning group did not (from *M* = 1.44 cm to *M* = 1.47 cm). However, since the power to detect such an effect was fairly low (<.29), this effect is likely not reliable. No other interactions were significant.

**Figure 3:**
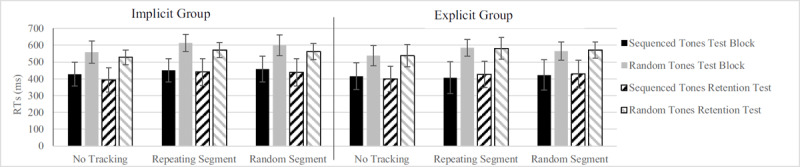
Reaction time results from the Test Block and Retention Test. Bars within each cluster show the RTs for sequenced and random tones per Group. Cluster on the x-axis represent the different conditions of the tracking task. Reactions to sequenced tones were significantly faster, but there was no difference in reaction times while tracking repeating or random segment. Error bars represent the standard deviation of the mean.

For the reaction time task there was a main effect of Tones, *F*(1, 30) = 178.73, *p* < .001, η_p_^2^ = .856 (sequenced tones *M* = 426 ± 79 ms; random tones *M* = 567 ± 55 ms) on RTs, showing that RTs to sequenced tones were lower than to random tones (***Figure 3***). We found significant dual-task costs, *F*(1.66, 30) = 15.268, *p* < .001, η_p_^2^ = .337, because RTs in the single-task condition (no segment) were shorter (*M* = 474 ± 67 ms), than in dual-task conditions. Participants responded to tones similarly fast while tracking both a repeating segment (*M* = 509 ± 67 ms) and a random segment (*M* = 505 ± 68 ms). Furthermore, there was a main effect of Test, *F*(1, 30) = 5.80, *p* = .022, η_p_^2^ = .16, because participants improved significantly from Test to Retention test (from 503 ms to 490 ms), and this effect was mainly due to the implicit learning group improving rather than the explicit learning group, as evidenced by a significant Test × Group interaction, *F*(1, 30) = 7.60, *p* = .010, η_p_^2^ = .202. While the implicit learning group lowered RTs from 518 ms to 489 ms, the explicit learning group slightly increased RTs from 488 ms to 490 ms. Lastly, we found a significant Tones × Segment interaction, *F*(2, 60) = 4.17, *p* = .020, η_p_^2^ = .122, which indicated that responding to random tones suffered more from dual-task conditions (ST = 540 ms and DT = 581 ms) than did responding to sequenced tones (ST = 409 ms and DT = 433 ms). No other interactions were significant.

#### Explicit knowledge interviews

None of the participants in the implicit learning group could verbalize explicit knowledge about the repeating middle segment during the first 5 probing questions. For question 6 (“There were three segments in the path, the first, the middle and at the last segment. One of these segments was always repeating? Did you notice?”) two participants said they noticed a repeating segment, however for question 7 (“Which segment was the repeating segment, the first, the middle or the last segment?”) only one of them correctly identified the middle one as repeating. Two participants said the first segment, seven said the middle segment, and eight said the last segment would repeat. All participants noticed the repeating pattern of the tones after the first trial. This may have caused a shift in prioritization to the tone task. If predictability influences prioritization, as argued above, this should have equalized prioritization effects through this factor if we assume that prioritization can also be influenced by predictability caused by implicit learning.

### DISCUSSION

The goal of the first experiment was to investigate the effect of predictability on dual tasks after single-task training. Predictability benefitted both tasks under dual-task conditions but this did not extend to the other task, mirroring the results of previous studies that the effect of predictability seems to be contained within the predictable task itself (Ewolds et al., 2017). Even though data suggested that resources were freed up in both tasks, participants did not exploit this to improve performance on the other task, as indicated by the absent effect of tones on RMSE. In line with Tombu and Jolicoeur (2003), who argued that task characteristics are decisive for resource allocation, it seems like “motor predictability” benefited visuomotor performance and auditory predictability benefited audiomotor performance only.

Furthermore, we found no dual-task costs in the tracking task, regardless of whether participants were tracking a random or predictable segment, and regardless of whether they tracked while responding to random or predictable tone sequences. For the auditory reaction time task, significant dual-task interference remained. A possible explanation for this is that the tracking task was trained for much longer.

Differences between implicitly and explicitly instructed participants were minor. During the test block the explicit learning group showed slightly better tracking performance on the predictable segment, but during the retention block the implicit learning group caught up and ended up better than the explicit learning group. The lower initial performance of the implicit learning group is not due to them entering the test phase with a lower amount of knowledge because at the end of the training phase both groups performed similarly (explicit learning group RMSE = 1.45 cm ± 0.23, implicit learning group RMSE = 1.46 cm ± 0.29). Nor can it be explained by a shift in priority by the implicit learning group from the tracking task to the reaction time task, the results of which followed a similar pattern as the tracking task, with initially worse performance but equally fast RTs on the retention test. Overall, this seems to suggest that the implicit learning group initially responded more poorly to dual-tasking than the explicit learning group, but then achieved similar RTs to those from the explicit learning group and even superior tracking performance.

The fact that we found no segment-specific learning effects during the training phase casts some doubt on whether segment effects in the test block reflected learning at all. In tracking studies learning effects have sometimes been ascribed to peculiarities of the repeating segment itself, which cannot have been the case in the current study as each participant received a unique repeating segment to practice. Nevertheless, segment effects might also be due to positioning, with performance usually being best at the start and deteriorating with time on task (Zhu et al., 2014). However, by placing the repeating segment in the middle and taking the average of the first segment and last segment as the performance metric for random tracking we should control for the time on task effect. A further possible measure to ensure that segment effects are due to learning only is by placing the repeating segment at the start, middle and end for different participants, which we did in Experiment 2.

In summary, we failed to show a redistribution of freed-up resources to both tasks during dual-tasking after both tasks are made predictable. We ruled out that this is due to prioritization effects due to only one task being predictable. A remaining reason for priority effects playing a role in these findings is that the tracking task was trained for a lot longer than the reaction time task. A possible way to let predictability effects spill over to the unpredictable task might therefore be through dual-task training, with both tasks being predictable, which we tested in Experiment 2. This would allow an equal amount of practice for both tasks, but a more important argument of why dual-task training might achieve spill-over effects is that we force participants to perform both tasks simultaneously from the beginning, which may result in the adoption of a more parallel task processing strategy where predictability in both tasks is exploited (Fischer & Plessow, 2015).

## EXPERIMENT 2

In the first experiment we found a task-specific effect of predictability on dual-task performance after practicing two predictable tasks separately. In the second experiment we investigate whether interference can be reduced through dual-task practice. This equalizes the amount of time spent on both tasks, ruling out the possibility that findings in Experiment 1 were due to the tracking task being practiced more. We kept the setup and experimental procedure of Experiment 1, see ***Figure 1***, but asked a new sample of participants to perform the training blocks under dual-task conditions, with the same predictable tracking and tone sequence that participants had learned (separately) in Experiment 1. The other difference compared to Experiment 1 is that the repeating segment of the tracking task now was placed either at the start, in the middle or at the end, equally distributed among participants within the implicit and explicit group, ensuring that learning effects are not due to peculiarities of tracking a segment in the middle position.

### MATERIALS AND METHODS

#### Participants

We tested 39 participants (29 female), the implicit learning group contained 19 participants (*M* = 24.0 ± 2.5 years) and the explicit learning group had 20 participants (*M* = 23.8 ± 2.4 years). The implicit group had only 19 participants because 1 participant discovered the repeating segment and was removed from analyses. None of the participants of Experiment 2 participated in Experiment 1. All participants had normal or corrected-to-normal vision and no reported neurological disorders. All participants gave informed consent prior to the start of the experiment and received remuneration of 20 € or course credit after completing the experiment. The research was approved by the local ethics committee. Experiment setup, task and display were identical to Experiment 1, except that we varied the position of the repeating segment in the tracking task between participants, for 12 participants the repeating segment was placed at the start, for 13 in the middle and for 10 at the end. The reason for this decision was that data from Experiment 1 showed that there might be an effect of segment position on performance, with generally better performance on the middle segment.

#### Procedure

The procedure of Experiment 2 differed from Experiment 1 in that participants performed the training of the tracking task always together with the auditory reaction time task. Participants were asked to give equal priority to both tasks.

#### Data analyses

Data analyses were similar to Experiment 1, with the exception that establishment of segment specific learning effects and learning of the always predictable reaction time task was done by applying one single ANOVA to the RMSEs and RTs, with within-subjects factors Block (4 training blocks), Segment (repeating segment vs. random segments), and between-subjects factors Group (implicit vs. explicit) and Segment position (start vs middle vs end).

### RESULTS

#### Acquisition of predictability-based knowledge

In the tracking task there was a main effect of Block as participants improved their tracking from Block 1 to 4, *F*(1.27, 36.75) = 13.98, *p* < .001, η_p_^2^ = .325 (Block 1: *M* = 1.97 ± 0.45 cm; Block: 4 *M* = 1.60 ± 0.43 cm), and a main effect of Segment, *F*(1, 29) = 9.86, *p* = .004, η_p_^2^ = .253, (repeating segment: *M* = 1.75 ± 0.42; random segment: *M* = 1.81 ± 0.39 cm), as performance on the repeating segment was better than on the random segments. There was no main effect of Group (implicit vs. explicit), *F*(1, 29) < 1, *p* = .589. There was no significant Block × Segment interaction, *F*(2.12, 61.40) = 1.21, *p* = .308, so tracking of the repeating segment did not improve more than tracking of the random segments. Also, the positioning of the repeating segment did not have a significant effect on tracking performance, *F*(2, 29) < 1, *p* = .530. In summary these effects show that knowledge specific to the repeating segment was acquired.

In the reaction time task there was also a main effect of Block *F*(1.57, 36.75) = 13.23, *p* < .001, η_p_^2^ = .313, (Block 1: *M* = 431 ± 95 ms; Block 4: *M* = 365 ± 97 ms), but no effect of Segment, *F*(1, 29) = 1.67, *p* = .207, or Group, *F*(1,29) < 1, *p* = .468.

#### Test Block and Retention Block

**Figure 4:**
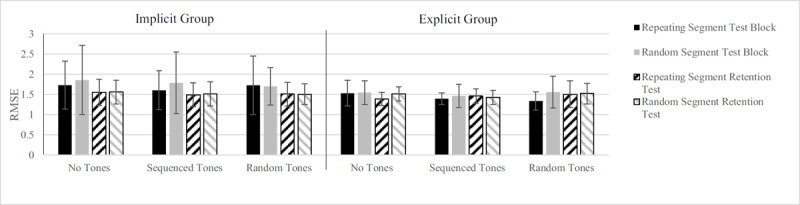
Tracking results from the Test Block and Retention Test in Root Mean Square Error (RMSE). Bars within a cluster show the tracking performance for repeating and random segments per group while the different cluster represent the conditions of the auditory task. We found no significant difference in tracking with sequenced or random tones. Error bars represent the standard deviation of the mean.

Analyses of the tracking task showed there was a main effect of Segment, *F*(1, 31) = 6.46, *p* = .016, η_p_^2^ = .172, (predictable segment: *M* = 1.53 ± 0.39 cm; random segment: *M* = 1.59 ± 0.41 cm), showing that tracking of predictable segments in the test block and retention test was significantly better than of random segments (***Figure 4***). There were no effects of Tones, *F*(2, 62) = 2.25, *p* = .114, or Group, *F*(1, 31) = 1.81, *p* = .188, so tracking performance did not differ between random, sequenced and no tone conditions, so there were no dual-task costs, and performance did not differ between the implicit and explicit learning group. Likewise, there were no significant effects of Test, *F*(1, 31) = 3.34, *p* = .077, η_p_^2^ = .097, or Test × Group interaction, *F*(1, 31) = 3.20, *p* = .084, η_p_^2^ = .093 . Finally, although we had no hypothesis for it, we found a significant Segment × Test effect, *F*(1, 31) = 5.11, *p* = .031, η_p_^2^ = .141, indicating that performance improved more on random (test block: *M* = 1.67 ± 0.29 cm; retention test: *M* = 1.51 ± 0.14 cm) compared to repeated segments (test block: *M* = 1.56 ± 0.31 cm; retention test: *M* = 1.49 ± 0.28) between the test block and the retention block.

**Figure 5:**
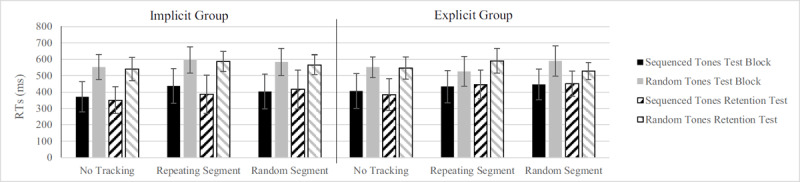
Reaction time results from the Test Block and Retention Test. Bars within each cluster show the RTs for sequenced and random tones per Group. Cluster on the x-axis represent the different conditions of the tracking task. There were no significant differences between reaction times while tracking a repeating or random segment. Error bars represent the standard deviation of the mean.

Looking at the reaction time task performance, there was a main effect of Tones, *F*(1, 27) = 147.24, *p* < .001, η_p_^2^ = .845 (predictable tones: *M* = 411 ± 72 ms; random tones: *M* = 563 ± 56 ms), as responding to predictable tones was faster than responding to random tones (***Figure 5***). No main effect of Group was found, *F*(1, 27) < 1, *p* = .675. We found a significant Segment effect, *F*(2, 54) = 10.01, *p* < .001, η_p_^2^ = .270, because RTs in the single task condition were shorter (*M* = 463 ± 67 ms), than in dual-task conditions (*M* = 499 ± 62 ms). No differences in performance between a repeating vs random segment were found. Furthermore, we found a significant Segment × Test × Group interaction, *F*(2, 54) = 5.49, *p* = .007, η_p_^2^ = .169, which meant that the implicit learning group improved from test block to retention block on the repeated segments (from *M* = 517 ms to *M* = 487 ms), the explicit learning group got worse on repeated segments (*M* = 481 ms to *M* = 518 ms). No other interactions were found.

### DISCUSSION

In Experiment 2 we found a task-specific effect of predictability on dual-task performance, similar to Experiment 1. Even after extensive dual-task practice, predictability in each task only improved performance on that task, ruling out the possibility that time on task was the reason for the task-specific predictability effects seen in Experiment 1. Importantly, the results of Experiment 2 also make it less likely that the task-specific predictability effects in Experiment 1 were due to a more serial processing mode as a consequence of having trained the tasks separately.

There was a consistent performance advantage of the predictable segment in Experiment 2, although, as in Experiment 1, we found no segment-specific training effects during the training blocks. It is likely that this is because the bulk of the segment-specific learning took place within the first training block. Alternative reasons for better performance of the repeating segment are segment-specific peculiarities and segment positioning but both were varied across participants and were shown to have no effect. We therefore conclude that learning did take place. Data of SRT task studies on whether a sequence can be learned while performing another task is conflicting but seems to point to difficulty being a key factor. Simpler, or first-order sequences (where each element in the sequence has a unique successor), may be learned under dual-task conditions whereas learning of second-order sequences is more susceptible to dual-task interference (Cohen et al., 1990; Curran & Keele, 1993; Keele et al., 2003; Nissen & Bullemer, 1987). In this connection our data support the idea that implicit learning of a tracking path is more similar to learning first-order sequences in SRT tasks, and that learning is not prevented by performing an additional task.

## GENERAL DISCUSSION

The goal of the current study was to investigate the effect of performing two predictable tasks simultaneously. We hypothesized that two predictable tasks reduce the need for resources for each task, and that both tasks would benefit from the overall reduction in capacity requirements and thereby reduce interference. Instead, we found that the effects of predictability only expressed themselves within each task, both after single- and dual-task practice. The main conclusion of the current study is that even after extensive dual-task practice the beneficial effects of predictability remain contained within each task.

### PREDICTABILITY AND PRIORITIZATION

In Experiment 1 we ruled out that task-specific predictability effects were due to differences in prioritization caused by predictability. In Experiment 2 we showed that differences in prioritization due to time on task were not the cause of the task-specific predictability effect, and that the increased time that tasks were practiced in the presence of each other also did not lead to a more even redistribution of resources. We hypothesized that a more even redistribution of resources might have occurred since a parallel mode of processing, which increases between-task interaction, might also ‘open up’ the task processing streams to benefit from resources freed-up in the other task. Usually, a more parallel processing mode can be demonstrated by the presence of backward crosstalk effects (Hommel, 1998; Janczyk et al., 2014; Navon & Miller, 1987), but this is difficult to establish with continuous tasks. On the other hand, the continuous nature of the tracking task makes it unlikely that the tracking task was ever ‘paused’ to process the auditory task. While not investigated in the current study, pauses in tracking were absent in Broeker et al. (2020)(under review), which used a similar paradigm as the current study, but see Netick and Klapp (1994) for a demonstration of hesitations in tracking during dual-task performance. These hesitations in tracking at the moment a tone is played would be an indication that there is some interference at the motor level, which might not show up in a summarized statistic of tracking performance over a whole trial, i.e. RMSE.

Even though prioritization effects were not due to time on task, the continuous nature of the tracking task may still have caused it to be prioritized. The distance between cursor and target serves as continuous feedback which is difficult to ignore. Such continuous feedback and the demand for continuous action is missing for the auditory task. Usually, instruction emphasizing equal importance of both tasks is used to prevent prioritization effects but the effectiveness of such instructions may be questioned. An equitable distribution of resources is difficult to achieve when there is no direct control over these resources. Indeed, in a study using similar tasks by Broeker et al. (2020, under review), a third of participants admitted to prioritizing the tracking task even though equal prioritization was instructed. Along with task features, a meta-analysis by Wickens et al. (2015) showed that in task-switching, task difficulty, salience and interest are all factors that can affect which tasks are prioritized. These factors may be at least partially individually determined, emphasizing the usefulness of an individual differences perspective and taking into account the effect of prioritization on resource allocation in dual-task performance (Broeker et al., 2018).

A further possible explanation for the effects of predictability might be due to the nature of the auditory task. The go/no-go task might call on significant inhibitory processes, which could lead to participants favoring a task-shielding strategy that might reduce interference but also prevents the spilling over of resources to the other task (Fischer & Plessow, 2015; Jong et al., 1995). The current experiments were not set up to detect different levels of involvement of inhibitory processes or task shielding, we can therefore not rule out that these processes played a role in the beneficial effects of predictability being contained within each task. Future studies could employ a task that is not reliant on inhibitory processes and measure the degree of task shielding to broaden the understanding of how predictability influences dual-task performance.

### SUSCEPTIBILITY TO DUAL-TASK COSTS

Data from both experiments show that the tracking task was not susceptible to dual-task performance decrements. Capacity theories predict the absence of dual-task costs when there is sufficient capacity available for both tasks and state that capacity can be increased by eliciting more effort from participants, which can be achieved by the dual-task situation itself (Kahneman, 1973; Tombu & Jolicœur, 2003). There are of course limits to this and with difficult tasks a capacity limit is quickly reached, leading to the interference effects found in most studies. It is hard to assess how difficult the tracking task was, and how consistent the level of difficulty was across participants. Nevertheless, it may be argued that tracking is a highly ideomotor compatible task, and these types of tasks have sometimes been shown not to be strongly influenced by dual-task interference (Greenwald, 2003), but see Lien et al. (2003) for a rebuttal.

Another reason for the absence of dual-task costs in tracking might have been that the tasks became intertwined so that participants were more used to ‘reacting with their foot while tracking a target’, than performing the tasks on their own, effectively representing or conceptualizing it as one task (Künzell et al., 2018), and possibly requiring costly response inhibition when only the tracking task was presented (Huestegge & Koch, 2014). The finding that speaks against integration of the two tasks, however, is that we found dual-task costs in the reaction time task. Task integration might be achieved by covarying both tasks, which should improve performance on both tasks (de Oliveira et al., 2017; Schmidtke & Heuer, 1997).

The finding that only the tracking task was free of dual-task interference is in line with the idea that a certain amount of capacity was saved for better performance on this task, with performance decrements only visible in the auditory task, in line with general capacity theories (Tombu & Jolicœur, 2003). As stated before, this prioritization of the tracking task is likely to be due to its continuous nature and not overridable through instructions, predictability effects, or time on the task. On the other hand, the multiple resource theory by Wickens (2008), more readily explains the task-specific effects of predictability by proposing that predictability only increases the available resources in the modalities of the specific task. These tasks draw from separate pools of resources that are not shared between tasks, so freeing up resources within these pools will only benefit the task itself, as they cannot be transferred to the resource pool of the other task. In summary, the difference in dual-task cost effects can be explained by prioritization or resource-saving effects acting on a general resource, whereas the effects of predictability staying within their respective tasks suggests that these tasks draw from separate pools of resources that are not shared between tasks. It should be noted that the general resource theory and the multiple resource theory are not incompatible with each other, indeed Wickens (2008) does not deny the existence of a general resource (or effort), and Kahneman (1973) noted that occurrences of structural interference could not always be accounted for by an undifferentiated resource pool. Indeed, an overview of the dual-tasking literature by Wahn and König (2017) shows that for studies combining object based tasks with spatial orientation tasks the evidence points towards a partial sharing of resources.

### LIMITATIONS

This study has some limitations. Firstly, it is difficult to assess to what extent an additional task takes up resources. Participants may be instructed to pay equal attention to two tasks but it is unclear what kind of problem-solving strategy is used. Do participants adopt a type of task-switching strategy where they alternatively focus on the tracking and tone task, and to what extent can participants divert their attention proportionally? These different strategies in solving dual-task problems are likely to be individually determined (Broeker et al., 2018). Furthermore, adding another task does not only take up attention, it also seems to alter the overall task structure itself, and it is not clear to what extent a dual-task consists of the two original single tasks or if task conceptualizations overlap (Künzell et al., 2018; Röttger et al., 2019). A promising avenue of research is therefore to find out how individuals differ in how they solve dual-task problems and how task conceptualizations might be influenced to optimize performance.

## DATA ACCESSIBILITY STATEMENT

Data of this project is available at: https://osf.io/f5h4g/.
